# Active Surveillance and Polymerase Chain Reaction (PCR) Between Two Passive Surveillance Periods Improve Detection and Characterization of Pleural Empyema: A 20-Year Study in Tijuana, Mexico

**DOI:** 10.7759/cureus.101369

**Published:** 2026-01-12

**Authors:** Erika Z Lopatynsky, Jaime A Rodriguez-Valencia, Enrique Chacón-Cruz

**Affiliations:** 1 Vaccinology and Infectious Diseases, Think Vaccines LLC, Houston, USA; 2 Pediatric Medicine, Hospital General de Tijuana, Tijuana, MEX; 3 Immunization Committee, Mexican Association of Pediatric Infectious Diseases, Mexico City, MEX

**Keywords:** active vs. passive surveillance, children, evidence-based decision-making, pleural empyema, pneumococcal conjugate vaccines, staphylococcus aureus

## Abstract

Pleural empyema (PE) surveillance was conducted at Tijuana General Hospital in Tijuana, Mexico, across three distinct periods: passive surveillance (2000-2005), active clinical surveillance with the incorporation of polymerase chain reaction (PCR)-based diagnostics (2005-2018), and a subsequent return to passive surveillance (2022-2024). The implementation of active surveillance coupled with molecular diagnostics substantially improved the detection of PE cases and the identification of causative pathogens, while also enabling the evaluation of the impact of pneumococcal conjugate vaccines (PCVs), including the 7-valent vaccine (PCV7; 2005-2012) and the 13-valent vaccine (PCV13; 2012-2018). This integrated approach provided a more accurate characterization of the etiologic spectrum of PE in children and adolescents, improved antimicrobial susceptibility profiling to better inform clinical management, and generated robust evidence supporting the high effectiveness of PCV13. Collectively, these findings highlight the value of sustained active surveillance and molecular diagnostics in strengthening evidence-based decision-making related to vaccine effectiveness, safety, and potential future vaccine implementation within national immunization programs.

## Introduction

Pleural empyema (PE) is a serious complication of bacterial pneumonia (BN) associated with high morbidity, prolonged hospitalization, potential sequelae, and, in some cases, death [1-3]. Among children beyond the neonatal period, the leading causative pathogens of both BN and PE are *Streptococcus pneumoniae, Staphylococcus aureus,* and *Haemophilus influenzae *[3-6].

At Tijuana General Hospital in Tijuana, Mexico, pediatric PE was monitored across three distinct phases: passive surveillance (2000-2005), active surveillance incorporating polymerase chain reaction (PCR) diagnostics (2005-2018), and a subsequent return to passive surveillance (2022-2024).

To date, no published studies have systematically compared passive and active surveillance for PE. The objective of this study is precisely to evaluate the impact of implementing active surveillance by comparing the detection rates and characterization of all variables associated with PE during periods of passive versus active surveillance, as well as after active surveillance was discontinued.

## Materials and methods

All children and adolescents aged three months to 16 years admitted to Tijuana General Hospital with a clinical diagnosis of pneumonia and chest radiography suggestive of PE underwent diagnostic thoracentesis. PE was defined by the presence of purulent pleural fluid, and patients meeting this criterion were included as cases in the study. Clinical and laboratory data were compiled in a Microsoft Excel database (Microsoft Corporation, Redmond, WA, USA), and descriptive statistical analyses were performed.

Because the study was conducted using standard-of-care procedures, the Institutional Review Board determined that formal approval was waived.

Three distinct surveillance periods were evaluated.

2000-2005: retrospective passive surveillance

Data were collected from hospital records of patients younger than 16 years diagnosed with PE.

2005-2018: prospective active surveillance

Active, prospective surveillance was initiated in the emergency department. Following pleural puncture, pleural fluid samples were immediately inoculated into radiometric broth media (BACTEC®, Becton Dickinson, Franklin Lakes, NJ, USA) and incubated at 37 °C with 5% carbon dioxide (CO₂). Final bacterial identification was performed using the Vitek/Microscan Vitek2® system (bioMérieux, Hazelwood, MO, USA).

For real-time PCR (RT-PCR), DNA was extracted from PF using the QIAamp DNA Blood Mini Kit (QIAGEN®, Shanghai, China) according to the manufacturer’s instructions. A 200 μL pleural fluid aliquot was processed, and DNA was eluted in 100 μL of TE buffer. RT-PCR was performed on the Mx3000P qPCR System (Stratagene®, La Jolla, CA, USA), targeting seven common bacterial pathogens associated with PE via specific gene markers: 16S (*Escherichia coli *​​​​​​(*E. coli)*), femA (*Staphylococcus aureus* (*S. aureus)*), hly (Listeria monocytogenes (*L. monocytogenes)*), ctrA (*Neisseria meningitidis* (*N. meningitidis)*), lytA (*Streptococcus pneumoniae* (*S. pneumoniae)*), bexA (*Haemophilus influenzae* (*H. influenzae)*), and cfb (Group B* Streptococcus*).

*S. pneumoniae* serotypes were identified by Quellung reaction (Statens Serum Institut®, Copenhagen, Denmark) and/or multiplex PCR following the CDC-recommended sequential method. Serotype-specific primers were validated with individual reference strains and subsequently tested against 5-10 additional clinical isolates to ensure broad detection within each serotype. For *S. aureus*, methicillin resistance was determined using 6 μg/mL oxacillin in Mueller-Hinton agar supplemented with 4% NaCl, or by PCR detection of the mecA gene.

2022-2024: passive surveillance reinstatement

Following the COVID-19 pandemic (2020-2022), when hospital admissions were limited to SARS-CoV-2 cases, passive surveillance for pediatric PE was reestablished.

## Results

2000-2005: retrospective passive surveillance

Between 2000 and 2005, 13 cases of PE were reported (2.6 cases per year). Pathogens were isolated by conventional culture in only four cases (30.7%): two *S. pneumoniae*, one *S. aureus*, and one *Streptococcus pyogenes* (*S. pyogenes*). Antimicrobial susceptibility testing was not performed for *S. pneumoniae* isolates. For* S. aureus* and *S. pyogenes*, testing by the disk diffusion method showed that the *S. aureus* strain was susceptible to oxacillin and glycopeptides, while the *S. pyogenes* strain was susceptible to penicillin.

2005-2018: prospective active surveillance

**Figure 1:**
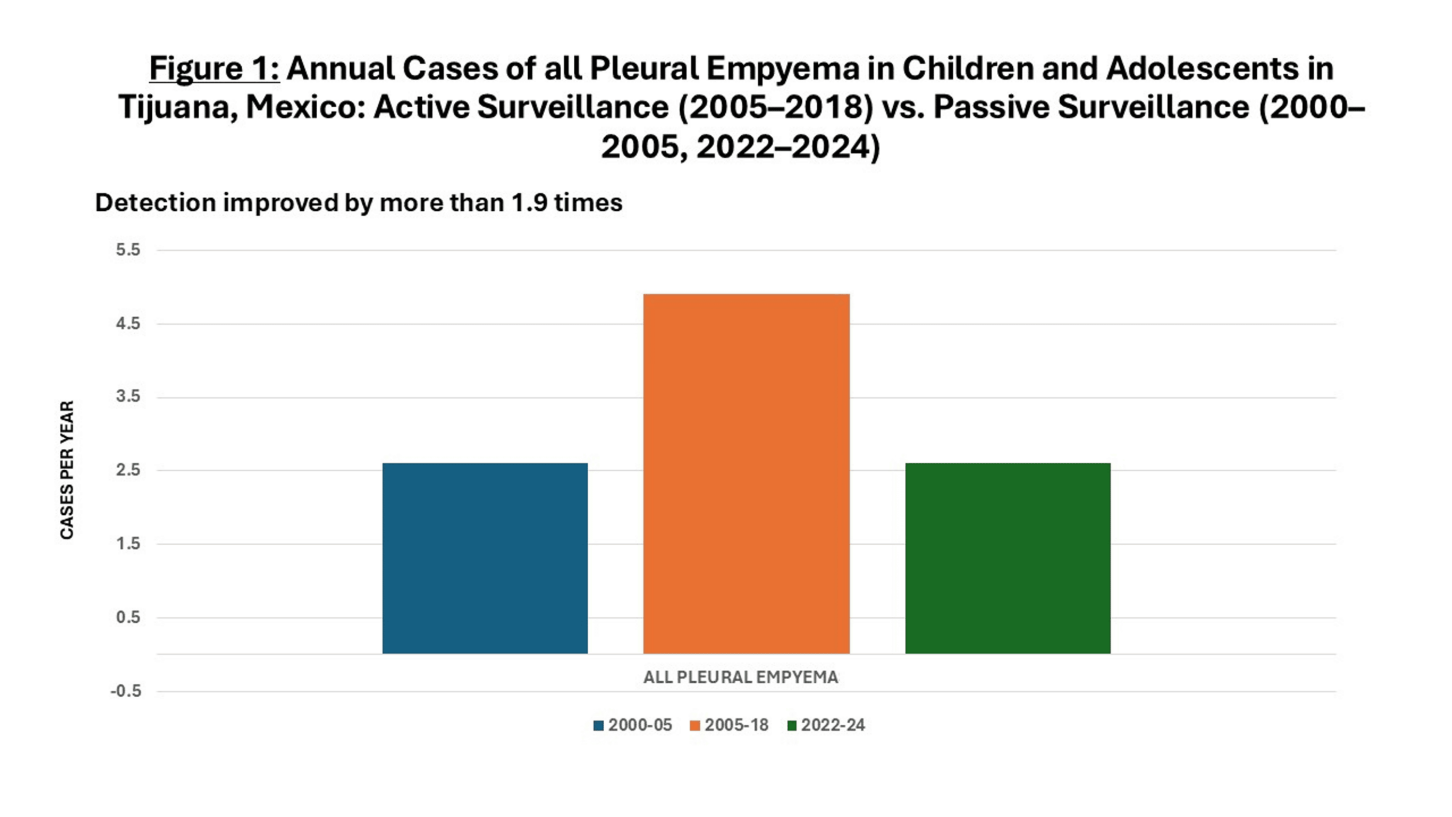
Annual Data of all Pleural Empyema Cases in Children and Adolescents in Tijuana, Mexico: Active Surveillance (2005–2018) vs. Passive Surveillance (2000–2005, 2022–2024)

**Figure 2:**
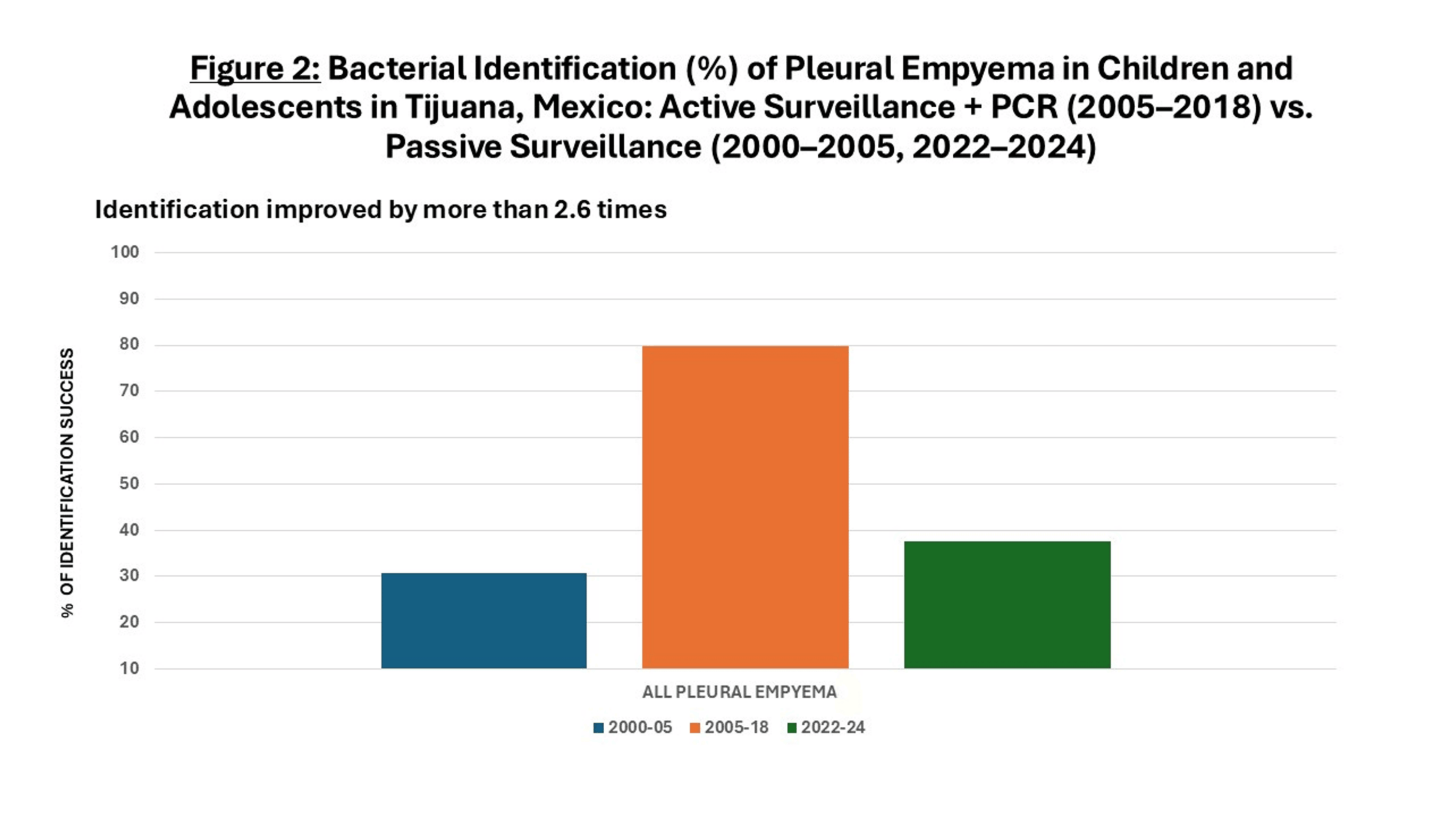
Bacterial Identification (%) of Pleural Empyema in Children and Adolescents in Tijuana, Mexico: Active Surveillance + PCR (2005–2018) vs. Passive Surveillance (2000–2005, 2022–2024) PCR: polymerase chain reaction

Between 2005 and 2018, 64 cases of PE were actively identified (4.9 cases per year), representing a 1.9-fold increase attributable to clinical active surveillance only compared with the 2000-2005 period (Figure 1). The addition of PCR to active surveillance enabled pathogen identification in 51 cases (79.7%), a 2.6-fold improvement in detection compared to the earlier period (Figure 2). The predominant organisms were *S.pneumoniae* (29; 56.8%), *S. aureus* (14; 27.4%), and *S. pyogenes* (3; 5.9%). Single cases were also attributed to *Peptostreptococcus sp.*, *Pseudomonas aeruginosa* (*P. aeruginosa), Klebsiella oxytoca* (*K. oxytoca), Streptococcus salivarius* (*S. salivarius),* and *Streptococcus milleri* (*S. milleri)*.

Detailed clinical, laboratory, imaging, treatment, and outcome data for each patient, as well as the impact of polysaccharide-protein pneumococcal conjugate vaccines (PCV7 and PCV13), have been previously published [7]. Briefly, that study reported PCV13 effectiveness of 53.8% against pneumococcal PE; however, an increase in PE caused by *S. aureus* was observed, predominantly due to methicillin-sensitive strains.

2022-2024: passive surveillance reinstatement

Between 2022 and 2024, eight cases of PE were reported (2.6 cases per year), representing a 1.9-fold decrease compared with the 2005-2018 period (Figure 1). Pathogens were isolated in only three cases (37.5%), reflecting a 2.1-fold reduction in bacterial identification relative to the prior period (Figure 2). The isolated organisms were *S.pneumoniae, S. aureus,* and *Klebsiella pneumoniae *(*K. pneumoniae)*, one of each, with no antimicrobial susceptibility data available.

## Discussion

As mentioned in the introduction, the objective of this study is precisely to evaluate the impact of implementing active surveillance by comparing the detection rates and characterization of all variables associated with PE during periods of passive versus active surveillance, as well as after active surveillance was discontinued.

For pneumococcal disease, multiple studies have demonstrated that active surveillance combined with molecular diagnostics (PCR) is crucial-not only for improving case detection across the spectrum of pneumococcal infections, but also for monitoring serotype dynamics following the introduction of PCVs. This approach enables a more accurate characterization of clinical outcomes, sequelae, morbidity, mortality, and the identification of other pathogens involved [8-15].

Particularly for PE, there are various publications of pediatric PE by implementing active surveillance.

In Germany, Forster et al. conducted a prospective study using PCR to evaluate the impact of prehospital antibiotic therapy on clinical outcomes and pathogen detection in 1,724 children with parapneumonic pleural effusion or empyema between 2010 and 2018. Their findings showed that while prehospital antibiotic administration significantly reduced bacterial pathogen detection by culture, it did not affect detection by PCR. Moreover, prehospital antibiotic use was associated with fewer infectious complications but did not influence the overall duration of illness [16].

In Turkey, pleural fluid samples from 156 children diagnosed with pneumonia complicated by empyema across 13 hospitals between 2010 and 2012 were collected via thoracentesis and tested for 14 *S. pneumoniae* serotypes/serogroups using a Bio-Plex multiplex antigen detection assay. The estimated serotype coverages for PCV7, PCV10, and PCV13 were 16.3%, 45.4%, and 60%, respectively. The authors concluded that active surveillance studies are essential to monitor shifts in *S. pneumoniae* serotypes causing empyema, in order to guide optimal pneumococcal vaccine selection [17]. Similar results were found in children with parapneumonic pneumonia in Italy and Japan [18, 19].

Our first publication in 2019 not only demonstrated an increase in the detection of PE cases but also provided enhanced insights into: (1) clinical, demographic, and patient outcomes; (2) the impact of PCV7 and PCV13 on pneumococcal serotype distribution and replacement; and (3) the contribution of pathogens other than *S. pneumoniae* [7]. Additionally, through active surveillance combined with molecular diagnostics (PCR), we observed the emergence of PE cases caused by *S. aureus.*

Although studies from the United States have not shown an increase in *S. aureus*-associated pneumonia following PCV13 implementation [20], several reports have documented a potential rise in *S. aureus*-related PE cases after the introduction of PCV13.

In Italy, Carloni et al. analyzed 43 pediatric cases and found a significant reduction in hospitalizations for necrotizing pneumonia (including PE) between the early (1.5/1000 admissions/year) and intermediate (0.35/1000 admissions/year) post-PCV13 periods (p = .001), followed by an increasing trend thereafter. *S. pneumoniae* remained the predominant pathogen in both periods (pre-PCV13: 11/18, 61%; post-PCV13: 13/25, 52%), with serotype 3 being the most frequent strain (pre-PCV13: 3/11, 27%; post-PCV13: 4/13, 31%). Although the overall etiology did not change substantially over time, most infections caused by *S. pyogenes* or *S. aureus* occurred during the post-PCV13 period [21].

In Germany, Lise et al. conducted a prospective study incorporating PCR in 1,447 children with parapneumonic pleural effusion or empyema between 2010 and 2017. Their findings showed an increase in *S. pyogenes* infections following PCV13 introduction, but no corresponding rise in *S. aureus* cases [22].

It is imperative to acknowledge the following limitations of this study. Prior to the implementation of active surveillance, the presence of purulent pleural fluid constituted the operational definition of PE at our institution. To ensure methodological consistency and comparability across all three study periods, we maintained this definition and therefore excluded non-purulent cases. We acknowledge that this approach may have resulted in the exclusion of some non-purulent PE cases; however, it allowed for consistent case ascertainment across study periods.

Additionally, incidence rates and corresponding confidence intervals could not be estimated because population-based denominators were unavailable, as Tijuana General Hospital does not serve a clearly defined catchment population. However, our study is the first to directly compare the benefits of implementing active surveillance and to demonstrate the substantial loss of essential epidemiologic information that occurs when this methodology is discontinued. At the time PCR was implemented during the active surveillance period (2005-2018), although sensitivity for certain pathogens may have been moderate (approximately 60-70%), the specificity of this molecular approach was close to 100%. However, despite these limitations, our study also has significant strengths.

Beyond pathogen identification and shifting serotype patterns, this study underscores that the true value of active surveillance lies in its ability to drive evidence-based decision-making for national immunization programs by complementing passive surveillance and clinical trials [22].

Robust surveillance systems-especially those strengthened with enhanced and molecular tools such as genotyping-generate the high-quality, timely, and comprehensive data needed to evaluate vaccine effectiveness, ensure safety, sustain public confidence, and guide rational vaccine introduction [23-26].

Importantly, these systems create a continuous quality-improvement loop, enabling health authorities to monitor disease epidemiology, rapidly detect vaccine-preventable threats, and refine immunization strategies as new evidence emerges. In this way, active surveillance becomes not only a technical activity but a foundational mechanism that informs clinical practice, shapes public health policy, and ultimately strengthens both routine immunization efforts and future pandemic preparedness [27-28].

## Conclusions

Active surveillance, incorporating immediate culturing and PCR sampling in the emergency department, doubled the detection rate of PE cases compared to passive surveillance. The combination of rapid culture and PCR enabled a 2.6-fold increase in bacterial identification, improved antimicrobial susceptibility testing, and supported more informed antibiotic decision-making.

Based on our observations, establishing active surveillance with molecular diagnostics also enabled evaluation of PCV effectiveness, with PCV13 demonstrating a strong impact in reducing pneumococcal PE, particularly those caused by vaccine-included serotypes.

As a surprising observational finding, PCV13 effectiveness for all pneumococcal PE was also associated with an increase in staphylococcal PE. Reinstating active surveillance with PCR is strongly recommended to ensure comprehensive pathogen detection and accurate epidemiologic monitoring and drive evidence-based vaccine policy-decision-making.
